# Income disparities in healthcare use remain after controlling for healthcare need: evidence from Swedish register data on psoriasis and psoriatic arthritis

**DOI:** 10.1007/s10198-017-0895-5

**Published:** 2017-05-19

**Authors:** Sofia Löfvendahl, Anna Jöud, Ingemar F. Petersson, Elke Theander, Åke Svensson, Katarina Steen Carlsson

**Affiliations:** 1 Department of Clinical Sciences Lund, Orthopedics, Faculty of Medicine, Lund University, Lund, Sweden; 2 Health Technology Assessment Skåne, Skåne University Hospital, Lund, Sweden; 3 Epidemiology and Register Centre South, Skåne University Hospital, Lund, Sweden; 4 Department of Rheumatology, Skåne University Hospital, Malmö, Sweden; 5 Department of Dermatology, Skåne University Hospital, Malmö, Sweden; 6 Department of Clinical Sciences, Malmö, Lund University, Skåne University Hospital, Malmö, Sweden; 7 Division of Occupational and Environmental Medicine, Lund University, Lund, Sweden; 8 Health Technology Assessment Skåne, Skåne University Hospital, Remissgatan 4, Wigerthuset, 221 85 Lund, Sweden

**Keywords:** Psoriasis, Psoriatic arthritis, Healthcare use, Socioeconomic factors, Disparities

## Abstract

We used a southern Swedish cohort of psoriasis (PSO) and psoriatic arthritis (PsA) patients and population-based referents (*N* = 57,800) to investigate the influence of socioeconomic and demographic factors on the probability of healthcare use and on healthcare costs when controlling for need as measured by PSO/PsA and common additional morbidities such as diabetes, depression and myocardial infarction. People with PSO/PsA were identified by ICD-10 codes in the Skåne Healthcare Register 1998–2007. Resource use and costs for years 2008–2011 were retrieved from the Skåne Healthcare Register and the Swedish Prescribed Drug Register, and socioeconomic data were retrieved from Statistics Sweden. After controlling for PSO/PsA and common additional morbidities, income, and to some extent education, had significant effects on the probability of five types of healthcare use. Overall, income showed a bell-shaped relationship to healthcare costs, with patients in income quintiles 2 and 3 having the highest mean annualized cost irrespective of model specification. Education did not have a significant effect in most specifications. Analyses including interaction effects indicated similarly higher costs across income quintiles in the PSO and PsA subgroups, though these cost differences were lower in magnitude for patients with PSO in quintile 5 and with PsA in quintile 1. In conclusion, our results show persistent socioeconomic disparities in healthcare use among a cohort of chronically ill patients and referents, even after controlling for the presence of PSO/PsA and common additional morbidities. These disparities persist even in a country with general healthcare coverage and low out-of-pocket payments.

**Electronic supplementary material:**

The online version of this article (doi:10.1007/s10198-017-0895-5) contains supplementary material, which is available to authorized users.

## Introduction

Equity in healthcare is recognized as an important policy issue in most Western countries [1], and many European healthcare systems have a guiding principle aiming at distributing healthcare according to need, often coupled with an organization that reduces the impact of the individual’s inability to pay for healthcare at the point of use.

Equity is often discussed in terms of fairness or justice, and health economists have written extensively on this subject [2, 3]. The theoretical literature offers a multitude of equity concepts and ways to study and measure equity in healthcare (see, e.g., [3–6]). One commonly used method for the analysis of equity in healthcare is to examine to what extent there is equal healthcare use for equal need in a healthcare system. In empirical work, need has to be operationalized into observable characteristics. In addition to age and sex, examples of such characteristics used in the literature are presence of indicators of morbidity or disability [7–11]. Variables such as education, income, employment status and ethnicity have, on the other hand, been cited as examples of factors that by themselves should not drive healthcare use, although in empirical analyses they may be a proxy for potential unobserved need [12–14].

Previous research on disparities in healthcare use across socioeconomic groups has focused on the effect of income after controlling for healthcare needs [1, 8, 10, 11, 14–18]. In a majority of the studies on equity in healthcare, researchers have based their analyses on a random sample of the adult general population [1, 8, 11, 14–16], and often the studies also share the feature of a reliance on self-reported information in the collection of data on healthcare need.

Another approach is to focus on narrower health conditions and to use register-based data to determine the presence of different diseases/conditions and healthcare use (see, e.g., [19, 20]). Patients who share specific diseases or conditions are more likely to have comparable healthcare needs. Moreover, the volume and type of healthcare services can also be expected to be more homogeneous. However, as pointed out by Doorslaer et al. [21], one drawback of this approach is that it comes with the price of losing a part of the system-wide perspective.

In this study, we used a register-based cohort from the Skåne region in southern Sweden consisting of patients with a physician-confirmed diagnosis of psoriasis (PSO) or psoriatic arthritis (PsA) and compared those patients with population-based referents free from PSO and PsA. The aim was to investigate the influence of income, education and country of birth on the probability of healthcare use and healthcare costs after controlling for healthcare need as measured by register-based information on the presence of the two specific chronic conditions PSO and PsA. In addition, we explored the robustness of the estimated coefficients to the introduction of three additional morbidities (metabolic, mental and circulatory diseases) that are common in the general population and which have a known increased risk among people with PSO and PsA. We hypothesized that patients with PSO/PsA would be more likely to use healthcare and have higher healthcare costs than population-based referents, even after controlling for additional comorbidities. We further hypothesized that education and income may have an influence on healthcare use for both patients and referents.

### Rationale and contribution

Though PSO and PsA had long been considered mild diseases and had received limited attention compared with more acute diseases such as heart disease and cancer, recent work has challenged this view [22]. PSO is a chronic inflammatory disease mainly affecting the skin and nails. In Sweden, the prevalence of psoriasis is about 1–3% [23]. Around 7–30% of the patients with PSO develop PsA, a chronic inflammatory disease that, in addition to affecting the skin, is manifested as pain, stiffness and swelling in and around the joints or in the back [23]. Many of those suffering from these diseases experience reduced health-related quality of life, functional limitation, pain, stigmatization and work disability [24]. As an added burden, there is evidence of an increased occurrence of other morbidities in these patient groups. Metabolic diseases such as diabetes and obesity, mental disorders such as schizophrenia and depression, and circulatory diseases such as myocardial infarction, hypertension and hyperlipidemia are examples of problems common in the Swedish general population [25], and have been cited as comorbidities in epidemiological studies among individuals with PSO and PsA [26–28], with an increased prevalence compared with the general population [29–31].

Studies have shown that patients with PSO and PsA use more healthcare and have more lost productivity than population-based referents free from these diseases [32, 33]. Yet little is known about the distribution of healthcare use in this cohort and to what extent socioeconomic status is associated with healthcare use for patients with POS/PsA.

By combining a study population of people with specific chronic diseases and a matched population-based referent cohort, we were able to explore the effect of non-need variables within the PSO and PsA groups, as well as to discern the impact of socioeconomic and demographic variables on healthcare use and costs for the overall study population. Besides increasing our understanding of healthcare spending for patients with PSO and PsA, this paper contributes to the existing empirical literature on disparities in healthcare use in a number of ways. Unlike many other studies that are largely based on self-reported data, we exclusively used individual-level longitudinal data from regional and national registers. This approach reduces biases of self-reports including recall bias and difficulties of disease categorization for lay people [34]. Our detailed data enabled analyses by type of service to explore utilization patterns across different healthcare service levels. Furthermore, we had 4 years of population-based data on healthcare use, which is a longer time window than normally seen in the empirical literature, and should therefore be less vulnerable to the influence of outliers or individual healthcare use variations over time. In our analyses, we explored the impact of two alternative measures of socioeconomic status, income and education, and one sociodemographic measure, country of birth. Finally, we used Cox proportional hazards regression for the estimation of the probability of healthcare use, to appropriately handle data in the longer than usual observation time window and also to account for possible censoring during the study period, an issue often overlooked in studies of the probability of healthcare use.

### The Swedish setting

Swedish healthcare is predominately tax-financed (91%), with user fees and private insurance covering 8 and 0.29% of the total healthcare expenditure, respectively [35]. A small user fee is paid by the patient until spending has reached approximately 112 € for healthcare contacts and 244 € for prescription drugs during a 12-month period (1 € = 9.03 SEK in 2011). After that, all out-of-pocket payment is waived during the rest of the 12-month window following the date of the first consultation or drug prescription in the period. The responsibility for providing healthcare in Sweden is decentralized to 21 regions of different sizes. Public and private providers offer healthcare, and both are predominantly tax-financed (see above). The usual path into healthcare is by a visit to a general practitioner, but patients can also access secondary care directly. As in a recent Swedish study on socioeconomic inequalities in drug utilization [11], we assumed that financial barriers to healthcare consumption exist but are low and have a comparatively small expected influence for a majority of patients. Still, some groups may be economically vulnerable — for example, retired people with only a basic pension (approximately 9500 € in median annual pension before tax). However, such low income is combined with eligibility for supplementary pension and housing supplement allowance. In our overall study population, 19% of individuals were over 65 years of age, and of these, around one quarter were in the lowest income quintile of the study.

## Methods and materials

### Study populations

We used individual data from a southern Swedish cohort consisting of patients with a physician-confirmed diagnosis of PSO or PsA at routine visits and population-based referents without either of these diseases. The patients in the cohort were identified by ICD-10 codes associated with PSO and PsA using information from a 10-year period, 1998–2007, in the Skåne Healthcare Register (SHR), which covers all healthcare used by the population (total *N* = 1.3 million 2015) in the Skåne region, which is in the southernmost part of Sweden. The SHR continuously registers all primary care, secondary outpatient care and inpatient care for residents in the Skåne region and includes information about age, sex, healthcare provider, date of consultation, and diagnostic codes according to ICD-10 and healthcare costs.

In this particular study, we included patients ≥ 19 years of age living in Skåne December 31, 2007. We created a population-based referent group from the Swedish Population Register by matching three referents on year of birth, sex and residential area for each included patient. Also, referents were resident in the Skåne region December 31, 2007, and were required to have no history of registered healthcare use associated with PSO or PsA in the SHR during 1998–2011.

### Description of study population

The characteristics of the cohort are shown in Table 1. A total of 14,450 patients fulfilled the inclusion criteria for PSO (*n* = 11,793) or PsA (*n* = 2657), and we had 43,350 referents. Using a chi-squared test for categorical data and a Student’s *t*-test for continuous data, we found small differences between the study groups. The PSO/PsA patients had marginally lower education and were born in a Nordic country to a greater extent than the referents. The characteristics of the PSO and PsA patients differed slightly; those with PsA were more likely to be female, young and born in a Nordic country. The majority of the individuals had at least one outpatient healthcare contact during the 4-year study period (2008–2011). Also, as many as 38 and 32% of the PSO/PsA and referent groups, respectively, registered at least one inpatient episode. As expected, PSO/PsA patients incurred higher mean annual healthcare costs during the study period than did the referents, and PsA patients incurred higher costs than PSO patients. Descriptive statistics for healthcare use and associated costs across socioeconomic and demographic variables for the study population are presented in Tables S1 and S2 in the Online Supplementary Material.

**Table 1:** Characteristics of the study population

	PSO/PsA patients	Referents	*p* value	PSO alone patients	PSO with PsA patients	*p* value
*N* ^a^	14,450	43,350		11,793	2657	
Women, *n* (%)^a^	7363 (51)	22,089 (51)	NA	5878 (50)	1485 (56)	<0.001
Age^a^						
Women, mean (SD) years	55 (18)	55 (18)	NA	55 (19)	55 (14)	0.17
Median (25th–75th; min–max)	56 (40–68)	56 (40–68)		56 (39–69)	57 (45–65)	
Men, mean (SD) years,	54 (17)	54 (17)	NA	54 (17)	55 (18)	0.01
Median (25th–75th; min–max)	55 (42–66)	55 (42–66)		55 (41–66)	55 (45–64)	
Age group, *n* (%)^a^			NA			<0.001
Age 20–64 years	9787 (68)	29,361 (68)		7896 (67)	1891 (71)	
Age ≥ 65 years	4663 (32)	13,989 (32)		3897 (33)	766 (29)	
Exit during follow-up, *n* (%)	1389 (9.6)	4169 (9.6)		1215 (10.3)	174 (6.5)	
Deaths	1020 (7.0)	2508 (5.8)		885 (7.5)	135 (5.1)	
Relocation out of Skåne	369 (2.6)	1661 (3.8)		330 (2.8)	39 (1.5)	
Metabolic disease, *n* (%)^c^	3909 (27)	8894 (21)	<0.001	3108 (26)	801 (30)	<0.001
Diabetes, type 1 or 2, *n* (%)^d^	1625 (11)	3187 (7)	<0.001	1302 (11)	323 (12)	0.103
Mental disorders, *n* (%)^e^	3291 (23)	7870 (18)	<0.001	2684 (23)	607 (23)	0.924
Psychiatric disease including schizophrenia and mood disorders, *n* (%)^f^	1429 (10)	3396 (8)	<0.001	1128 (10)	301 (11)	0.006
Circulatory disease, *n* (%)^g^	5919 (41)	14,651 (31)	<0.001	4735 (40)	1184 (45)	<0.001
Ischemic heart disease including angina and myocardial infarction, *n* (%)^h^	1122 (13)	4277 (10)	<0.001	1486 (13)	336 (13)	0.950
Education, *n* (%)^b^			0.02			0.10
Low (≤9 years)	3861 (27)	11,134 (26)		3134 (27)	727 (27)	
Medium (10–12 years)	8148 (56)	23,035 (53)		6606 (56)	1542 (58)	
High (≥12 years)	2029 (14)	7590 (18)		1687 (14)	342 (13)	
Missing	412 (3)	1591 (4)		366 (3)	46 (2)	
Income, *n* (%)^b^			<0.001			0.27
Quintile 1 (Low)	2558 (18)	8789 (20)		2107 (18)	451 (17)	
Quintile 2	3056 (21)	8268 (19)		2458 (21)	598 (23)	
Quintile 3 (Median)	3048 (21)	8282 (19)		2467 (21)	581 (22)	
Quintile 4	2861 (20)	8470 (20)		2350 (20)	511 (19)	
Quintile 5 (High)	2641 (18)	8691 (20)		2161 (18)	480 (18)	
Missing	286 (2)	850 (2)		250 (2)	36 (1)	
Country of birth, *n* (%)^b^			<0.001			<0.001
Nordic	13,166 (91)	37,332 (86)		10,664 (90)	2502 (94)	
Non-Nordic	1282 (9)	6010 (14)		1127 (10)	155 (6)	
Missing	2 (0)	8 (0)		2 (0)	–	
Any healthcare contact, *n* (%)	14,318 (99)	41,663 (96)	<0.001	11,668 (99)	2650 (99.7)	<0.001
Primary care physician contact, *n* (%)^i^	13,349 (92)	37,476 (86)	<0.001	10,869 (92)	2480 (93)	0.039
Secondary care physician contact, *n* (%)^i^	13,138 (91)	35,578 (82)	<0.001	10,579 (90)	2559 (96)	<0.001
Inpatient care contact, *n* (%)^i^	5502 (38)	13,778 (32)	<0.001	4408 (37)	1094 (41)	<0.001
Primary care, non-physician contact, *n* (%)^j^	12,071 (84)	33,853 (78)	<0.001	9768 (83)	2303 (87)	<0.001
Secondary care, non-physician contact, *n* (%)^j^	9350 (65)	22,048 (51)	<0.001	7363 (62)	1987 (75)	<0.001
Mean (SD) total annual healthcare costs during follow-up in 2008–2011 period^k^	4974 (8970)	3261 (7082)	<0.001	4500 (8845)	7061 (9217)	<0.001

*NA* not applicable, since PSO/PsA patients and referents are matched on sex, age and residential area

^a^At the end of 2007, ^b^Reported in 2008

^c^Endocrine, nutritional and metabolic diseases according to ICD-10 classification system (E00–E90)

^d^Common disease within the ICD-10 chapter E: diabetes type 1 or type 2 (E10, E11)

^e^Mental and behavioral disorders according to ICD-10 classification system (F00–F99)

^f^Common disease within the ICD-10 chapter F: psychiatric disease including schizophrenia and mood disorders (F20–F29, F30–F39)

^g^Diseases of the circulatory system according to ICD-10 classification system (I00–I99)

^h^Common disease within the ICD-10 chapter I: ischemic heart disease including angina and myocardial infarction (I20–I25) and stroke (I61, I63, I64)

^i^At least one physician contact during follow-up in 2008–2011 period

^j^At least one visit with other healthcare personnel during follow-up in 2008–2011 period

^k^Total healthcare costs include costs due to drug use in addition to costs due to visits within primary care, secondary outpatient care and inpatient care. Includes individuals with any healthcare contact during 2008–2011 (*N* = 55,981). All costs are expressed in euros at 2011 prices

We used Stata v13.0 software (StataCorp, College Station, Texas, USA) for all the statistical analyses.

### Study period of outcome variables

Data on healthcare use and associated costs for PSO/PsA patients and the referent cohort were observed for a 4-year period (2008–2011), in order to reduce the impact of variations in short-term healthcare use on the results. The data were retrieved from Swedish national and regional registers and were linked together by personal identification numbers.

### Outcome variables

First, we analyzed factors affecting the risk of use of five types of healthcare utilization services during the study period 2008–2011: primary care (physician and non-physician), secondary outpatient care (physician and non-physician) and inpatient care. Non-physician contacts were, for instance, visits to a nurse, physiotherapist or occupational therapist. Second, we analyzed the average annual costs of the healthcare use, including the cost of prescription drugs for people with service during the 2008–2011 period (99% of people with PSO/PsA and 96% of referents).

Healthcare use and costs were collected from the SHR. To attach monetary value to each individual’s healthcare consultation and inpatient care, we used individual center-specific unit costs from the SHR.

Data on the cost of prescription drugs were collected from the Swedish Prescribed Drug Register (SPDR) at the National Board of Health and Welfare, a national individual-level data register, in which all dispensed prescribed drugs to the entire Swedish population are registered except for drugs given in hospitals [36]. The SPDR registers the total cost as the pharmacy wholesale price, which includes the cost paid by the patient and the subsidy paid by the healthcare region. We calculated the mean annualized cost for all healthcare use including drug use per patient over the 2008–2011 study period and adjusted the observation time for drop-outs resulting from relocation from the region or from death.

### Explanatory variables

We retrieved socioeconomic (education and income) and demographic (country of birth) information from the register-based individual-level data in the Longitudinal Integration Database for Health Insurance and Labour Market Studies (the LISA database) at Statistics Sweden. We used socioeconomic and demographic information for 2008, the first year of the study period.

#### Education

Level of education was defined as the highest achieved level of education, and three groups were defined: “Low” = 0–9 years, “Medium” = 10–12 years and “High” = 13 or more years. The group “Medium” was used as the reference category.

#### Income

Individualized disposable household incomes (including wage, transfers and taxes) were categorized into five income quintiles for all individuals in the study population. The third income quintile (“Median”) was used as the reference category.

#### Country of birth

The variable country of birth was split into two groups: “Nordic origin” = subject and both parents born in the Nordic countries (Sweden, Denmark, Norway, Iceland and Finland), and “non-Nordic origin” = the subject or at least one parent born outside the Nordic countries. Born in the Nordic countries was used as the reference category.

#### PSO, PsA and additional morbidities

We used the presence of PSO and PsA (PSO with additional manifestations from the joints) as the primary healthcare need variables. We also used additional morbidity variables. As mentioned previously, metabolic, mental and circulatory diseases are common in the Swedish general population, but they are also common morbidities among individuals with PSO and PsA.

The presence of these morbidities in any individual was identified as having at least one healthcare contact plus a physician-confirmed diagnosis (main or secondary) of a metabolic (ICD-10 chapter E — diagnostic codes E00–E90), mental (ICD-10 chapter F — codes F00–F99) or circulatory disease (ICD 10 chapter I — codes I00–I99) listed in the SHR during the study period. Table 1 presents the proportions of individuals with a contact within the three ICD-10 diagnostic chapters, as well as well-known subgroups within these broader groups. The reason for controlling for the full code range in the three chapters, and not for specific diagnostic codes, was that we wanted to encompass as much as possible of the underlying morbidity.

### Empirical strategy

The standard two-part framework for handling over-representation of zero use of healthcare is less relevant for our data, since they cover 4 years and hold few non-users. Moreover, it is a large data set (*N* = 57,800), and it has been argued that ordinary least squares regression is appropriate [37]. In addition, estimating the overall probability of use with so few non-users is not ideal, and in the model specifications with additional morbidities, all individuals with additional morbidities had at least some healthcare costs. We formulated two overall research questions to investigate healthcare use disparities when controlling for PSO, PsA and additional morbidities. First, what is the influence of socioeconomic variables on at least some use of five types of healthcare services? Second, what is the influence of socioeconomic variables on annualized healthcare costs among healthcare users? The rationale for this strategy was that there is reason to believe that the impact of socioeconomic factors differs between the initial treatment contact and the amount of care consumed [9, 10, 13, 38, 39]. The restriction of the sample to positive users was relaxed in a sensitivity analysis reported in the Online Supplementary Material.

Equation 1 presents a linear version of our empirical model of the form variable (coefficient): PSO/PsA diseases (*β*_1_), metabolic disease (*β*_2_), mental disorder (*β*_3_), circulatory disease (*β*_4_), education (*β*_5_), income (*β*_6_), born within/outside a Nordic country (*β*_7_) and error term (*ε*). The same set of variables were included in (1) Cox regressions of the decision to use healthcare or not at any given time-point (*y* = 0/1), and (2) semi-logarithmic linear regressions of the logarithm of mean annualized healthcare costs [*y* = ln(cost)].

1 $$y = \alpha + \beta_{1} {\text{PSO}}/{\text{PsA}} + \beta_{2} {\text{Metabolic}} + \beta_{3} {\text{Mental}} + \beta_{4} {\text{Circulatory}} + \beta_{5} {\text{Education}} + \beta_{6} {\text{Income}} + \beta_{7} {\text{Country of Birth }} + \varepsilon$$

We modified Eq. 1 by separately adding alternative interaction terms: (1) PSO/PsA*Metabolic, (2) PSO/PsA*Mental, (3) PSO/PsA*Circulatory, (4) PSO/PsA*Education, (5) PSO/PsA*Income, and (6) PSO/PsA*Country of Birth.

The rationale for including interactions between PSO/PsA and the other explanatory variables was to explore the potential variability of the size of the estimated effects within different strata of these variables for PSO/PsA patients in addition to the direct effect of each variable. Combinations of base and interaction effects were explored in six models, as shown in Table 2.

**Table 2:** Versions of Eq. 1

Model	Included explanatory variables
Model 1	PSO/PsA, Education, Income and Country of birth
Model 2	PSO/PsA, Metabolic disease, Mental disorder, Circulatory disease, Education, Income and Country of birth
Model 3	PSO/PsA, Metabolic disease, Mental disorder, Circulatory disease, Education, Income, Country of birth and interaction term PSO/PsA*morbidities
Model 4	PSO/PsA, Metabolic disease, Mental disorder, Circulatory disease, Education, Income, Country of birth and interaction term PSO/PsA*Education
Model 5	PSO/PsA, Metabolic disease, Mental disorder, Circulatory disease, Education, Income, Country of birth and interaction term PSO/PsA*Income
Model 6	PSO/PsA, Metabolic disease, Mental disorder, Circulatory disease, Education, Income, Country of birth and interaction term PSO/PsA*Country of Birth

We used Cox proportional hazards regression, with days to visit as the time variable, to analyze factors affecting the probability of healthcare use. This method was used because it accounts for differences in observation time resulting from censoring. Observations were censored at the date of death, relocation out of the Skåne region or the end of the study period (December 31, 2011). The results are presented as hazard ratios (HR) with 95% confidence intervals (CI). To verify the proportional hazards assumption in the Cox model, we plotted the relative hazards over time for each categorical variable. By visual inspection, all of the variables were considered to meet the proportional hazards assumption. In the analysis of the mean annualized healthcare costs, we used a semi-logarithmic ordinary least squares (OLS) regression to handle the skewed distribution of healthcare costs. Coefficients for categorical variables are interpreted as the percentage difference compared with the reference category.

Both types of regressions accounted for the matching variables. The Cox model was stratified by each “pair” of individuals with PSO/PsA and their matched referents [40]. The baseline hazard was accordingly allowed to vary between strata that captured age, sex and residential area. The semi-logarithmic model treated “pairs” of persons with PSO/PsA and referents as a fixed number of strata and included them in the regression as an absorbing categorical factor [41].

### Ethics

This study was conducted according to the Declaration of Helsinki and approved by the Regional Ethical Review Board in Lund, Sweden (Dnr 301/2007, Dnr 406/2008 and Dnr 2012/359).

## Results

### Healthcare use

Model 1 in Tables 3, 4, and 5 shows the hazard ratios and 95% confidence intervals for primary care use, secondary care use, and inpatient care use across PSO/PsA and socioeconomic/demographic variables. In Model 2, we also controlled for additional morbidity variables, i.e., metabolic, mental and circulatory diseases.

**Table 3:** Hazard ratios (HR) and 95% confidence intervals (CI) for the association of primary care use (at least one visit) and presence of PSO/PsA, comorbidities, and socioeconomic and demographic factors during follow-up in the 2008–2011 period

Variable^a^	Primary care—physician				Primary care—other healthcare personnel^b^			
	Model 1		Model 2		Model 1		Model 2	
	HR	95% CI	HR	95% CI	HR	95% CI	HR	95% CI
Presence of PSO/PsA								
No presence (Ref.)								
PSO	1.25***	1.22–1.29	1.19***	1.16–1.22	1.20***	1.17–1.23	1.14***	1.11–1.17
PsA	1.29***	1.22–1.36	1.20***	1.14–1.27	1.33***	1.25–1.41	1.23***	1.16–1.31
Metabolic disease^c, d^			1.33***	1.29–1.37			1.40***	1.35–1.45
Mental disorder^c, d^			1.49***	1.44–1.54			1.24***	1.20–1.28
Circulatory disease^c, d^			1.53***	1.49–1.59			1.55***	1.50–1.60
Education^¤^								
0–9 years	1.04***	1.01–1.07	1.02	0.99–1.05	1.00	0.97–1.04	0.98	0.95–1.01
10–12 years (Ref.)								
>12 years	0.83***	0.81–0.86	0.86***	0.83–0.89	0.92***	0.88–0.95	0.95***	0.91–0.98
Income^c^								
Quintile 1 (Low)	0.81***	0.78–0.85	0.81***	0.77–0.84	0.84***	0.81–0.88	0.84***	0.81–0.88
Quintile 2	0.95**	0.92–0.99	0.92***	0.89–0.96	0.98	0.94–1.02	0.96*	0.92–1.10
Quintile 3 (Ref.)								
Quintile 4	0.89***	0.86–0.93	0.93***	0.90–0.97	0.93***	0.89–0.96	0.97	0.93–1.01
Quintile 5 (High)	0.84***	0.81–0.88	0.90***	0.86–0.94	0.86***	0.82–0.89	0.91***	0.87–0.95
Born outside a Nordic country^c^	1.01	0.98–1.06	1.02	0.98–1.06	1.04**	1.00–1.09	1.04**	1.00–1.09
Observations^e^	55,771		55,771		55,783		55,783	

*** *p* < 0.001, ** *p* < 0.05, * *p* < 0.1

^a^The Cox model was stratified by each “pair” of individuals with PSO/PsA and their matched referents. The baseline hazard was accordingly allowed to vary between strata that captured age, sex and residential area. The matching variables are therefore omitted from the explanatory variable list

^b^Other = nurse, physiotherapist, occupational therapist, etc.

^c^Reference categories (Ref.) are referents, no morbidity, 10–12 years of education, income quintile 3 and born in a Nordic country. Ref. = 1

^d^Metabolic disease = ICD-10 group E00–E90, mental disorder = ICD-10 group F00–F99, circulatory disease = ICD-10 group I00–I99

^e^Observations with entry and exit on the same day are not included in the analysis

**Table 4:** Hazard ratios (HR) and 95% confidence intervals (CI) for the association of secondary care use (at least one visit) and presence of PSO/PsA, comorbidities, and socioeconomic and demographic factors during follow-up in the 2008–2011 period

Variable^a^	Secondary care—physician				Secondary care—other healthcare personnel^b^			
	Model 1		Model 2		Model 1		Model 2	
	HR	95% CI	HR	95% CI	HR	95% CI	HR	95% CI
Presence of PSO/PsA								
No presence (Ref.)								
PSO	1.41***	1.37–1.45	1.35***	1.31–1.39	1.41***	1.36–1.45	1.33***	1.28–1.37
PsA	2.22***	2.09–2.36	2.12***	2.00–2.25	2.07***	1.95–2.21	1.93***	1.81–2.06
Metabolic disease^c, d^			1.26***	1.22–1.30			1.68***	1.62–1.75
Mental disorder^c, d^			1.50***	1.46–1.56			1.50***	1.44–1.55
Circulatory disease^c, d^			1.42***	1.38–1.47			1.56***	1.51–1.62
Education^c^								
0–9 years	0.96**	0.93–0.99	0.94***	0.91–0.97	0.98	0.94–1.01	0.95***	0.91–0.98
10–12 years (Ref.)								
>12 years	0.97	0.93–1.00	1.00	0.97–1.04	1.01	0.96–1.05	1.05**	1.01–1.10
Income^c^								
Quintile 1 (Low)	0.87***	0.83–0.91	0.86***	0.83–0.90	0.94***	0.89–0.98	0.94***	0.89–0.98
Quintile 2	0.99	0.95–1.03	0.96*	0.92–1.00	1.06***	1.02–1.11	1.04	0.99–1.09
Quintile 3 (Ref.)								
Quintile 4	0.93***	0.90–0.97	0.97	0.93–1.01	0.86***	0.82–0.90	0.91***	0.87–0.96
Quintile 5 (High)	0.92***	0.88–0.96	0.97	0.93–1.01	0.82***	0.78–0.86	0.89***	0.85–0.94
Born outside a Nordic country^c^	1.03	0.99–1.07	1.03	0.98–1.06	1.04*	1.00–1.09	1.03	0.98–1.08
Observations^e^	55,744		55,744		55,785		55,785	

*** *p* < 0.001, ** *p* < 0.05, * *p* < 0.1

^a^The Cox model was stratified by each “pair” of individuals with PSO/PsA and their matched referents. The baseline hazard was accordingly allowed to vary between strata that captured age, sex and residential area. The matching variables are therefore omitted from the explanatory variable list

^b^Other = nurse, physiotherapist, occupational therapist, etc.

^c^Reference categories (Ref.) are referents, no morbidity, 10–12 years of education, income quintile 3 and born in a Nordic country. Ref. = 1

^d^Metabolic disease = ICD-10 group E00–E90, mental disorder = ICD-10 group F00–F99, circulatory disease = ICD-10 group I00–I99

^e^Observations with entry and exit on the same day are not included in the analysis

**Table 5:** Hazard ratios (HR) and 95% confidence intervals (CI) for the association of inpatient care use (at least 1 day) and presence of PSO/PsA, comorbidities, and socioeconomic and demographic factors during follow-up in the 2008–2011 period

Variable^a^	Inpatient care			
	Model 1		Model 2	
	HR	95% CI	HR	95% CI
Presence of PSO/PsA				
No presence (Ref.)				
PSO	1.23***	1.18–1.28	1.12***	1.08–1.17
PsA	1.49***	1.38–1.61	1.33***	1.22–1.44
Metabolic disease^b, c^			1.49***	1.43–1.56
Mental disorder^b, c^			1.71***	1.63–1.78
Circulatory disease^b, c^			2.52***	1.38–1.47
Education^b^				
0–9 years	1.09***	1.04–1.13	1.05**	1.00–1.10
10–12 years (Ref.)				
>12 years	0.95**	0.90–1.00	1.03	0.97–1.09
Income^b^				
Quintile 1 (Low)	0.99	0.94–1.05	0.98	0.92–1.04
Quintile 2	1.10***	1.04–1.16	1.05*	0.99–1.12
Quintile 3 (Ref.)				
Quintile 4	0.81***	0.76–0.86	0.86***	0.81–0.92
Quintile 5 (High)	0.75***	0.71–0.80	0.84***	0.78–0.89
Born outside a Nordic country^b^	0.94**	0.88–1.00	0.91***	0.86–0.97
Observations^d^	55,744		55,744	

*** *p* < 0.001, ** *p* < 0.05, * *p* < 0.1

^a^The Cox model was stratified by each “pair” of individuals with PSO/PsA and their matched referents. The baseline hazard was accordingly allowed to vary between strata that captured age, sex and residential area. The matching variables are therefore omitted from the explanatory variable list

^b^Reference categories (Ref.) are referents, no morbidity, 10–12 years of education, income quintile 3 and born in a Nordic country. Ref. = 1

^c^Metabolic disease = ICD-10 group E00–E90, mental disorder = ICD-10 group F00–F99, circulatory disease = ICD-10 group I00–I99

^d^Observations with entry and exit on the same day are not included in the analysis

Consistent with expectations, in Model 1 the probability of visiting a physician or non-physician professional was significantly associated with PSO and PsA across all healthcare levels, with the most pronounced hazard ratio for physician visits in secondary care for those with PsA (HR 2.22, 95% CI 2.09–2.36) (Table 4). When including additional morbidity variables (Model 2), PSO/PsA remained significantly associated with healthcare use, but to a lesser extent than in Model 1. Metabolic, mental and circulatory morbidities were highly associated with a healthcare visit, sometimes even more than PSO/PsA, at all healthcare levels. Overall, the association was most pronounced for circulatory disease.

In Model 1, low education (0–9 years) was consistently associated with a higher probability of primary care use (Table 3) and inpatient care use (Table 5), while the reverse was observed for secondary outpatient care use (Table 4), although the association was only significant for physician visits. When adding the additional morbidity variables (Model 2), the significant effect of low education disappeared for physician primary care use. High education (>12 years) was associated with a lower probability of primary care use, both with and without the additional need variables added (Table 3).

**Table 6:** Linear regression of factors influencing mean annual healthcare costs during 2008–2011 (costs are in logarithm form)

Variables^a^	Model 1		Model 2		Model 3		Model 4		Model 5		Model 6	
	*β*	95% CI	*β*	95% CI	*β*	95% CI	*β*	95% CI	*β*	95% CI	*β*	95% CI
Presence of PSO/PsA												
No presence (Ref.)												
PSO^b^	0.46***	0.43–0.49	0.36***	0.33–0.38	0.48***	0.45–0.52	0.39***	0.35–0.43	0.26***	0.20–0.32	0.35***	0.32–0.38
PsA	1.06***	1.00–1.12	0.92***	0.87–0.98	1.26 ***	1.18–1.34	0.98***	0.91–1.06	0.87***	0.75–0.99	0.93***	0.87–0.99
Metabolic disease^b, c^			0.53***	0.50–0.56	0.57***	0.53–0.60	0.53***	0.50–0.56	0.53***	0.50–0.57	0.53***	0.50–0.56
Mental disorder^b, c^			0.71***	0.68–0.74	0.76***	0.72–0.80	0.71***	0.68–0.74	0.71***	0.68–0.74	0.71***	0.68–0.74
Circulatory disease^b, c^			0.77***	0.74–0.80	0.84***	0.80–0.87	0.77***	0.74–0.80	0.77***	0.74–0.80	0.77***	0.74–0.80
PSO/PsA and additional morbidities												
PSO and metabolic					−0.08**	−0.15 to 0.00						
PsA and metabolic					−0.26***	−0.39 to 0.12						
PSO and mental					−0.14***	−0.21 to 0.06						
PsA and mental					−0.32***	−0.46 to 0.19						
PSO and circulatory					−0.20***	−0.27 to 0.14						
PsA and circulatory					−0.44***	−0.57 to 0.32						
Education^b^												
0–9 years	0.06***	0.03–0.10	0.01	−0.02 to 0.04	0.01	−0.02 to 0.07	0.05***	0.02–0.09	0.01	−0.02 to −0.04	0.02	−0.01 to −0.05
10–12 years (Ref.)												
>12 years	−0.09***	−0.12 to −0.05	−0.02	−0.06 to −0.01	−0.02	−0.05 to 0.02	−0.02	−0.06 to −0.02	−0.02	−0.06 to 0.06	−0.02	−0.06 to 0.01
PSO/PsA and education^d^												
PSO and 0–9 years							−0.12***	−0.18 to−0.05				
PSO and >12 years							0.00	−0.08 to0.09				
PsA and 0–9 years							−0.22***	−0.39 to −0.10				
PsA and >12 years							−0.01	−0.35 to −0.09				
Income^b^												
Quintile 1 (Low)	−0.05**	−0.09 to −0.01	−0.07***	−0.11 to −0.03	−0.07***	−0.11 to 0.03	−0.07***	−0.11 to −0.03	−0.09***	−0.14 to −0.05	−0.07***	−0.11 to −0.03
Quintile 2	0.12***	0.07–0.16	0.06***	0.02–0.10	0.06***	0.02–0.10	0.06***	0.02–0.10	0.06***	0.01–0.10	0.06***	0.02–0.10
Quintile 3 (Ref.)												
Quintile 4	−0.19***	−0.23 to −0.15	−0.10***	−0.13 to −0.06	−0.10***	−0.13 to 0.06	−0.09***	−0.13 to −0.06	−0.14***	−0.19 to −0.10	−0.10***	−0.13 to −0.06
Quintile 5 (High)	−0.32***	−0.37 to −0.28	−0.19***	−0.23 to −0.15	−0.18***	−0.22 to 0.14	−0.18***	−0.22 to −0.14	−0.24***	−0.28 to −0.19	−0.19***	−0.23 to −0.15
PSO/PsA and income^e^												
PSO and income Q1									0.11**	0.02–0.21		
PSO and income Q2									−0.00	−0.09–0.09		
PSO and income Q4									0.20***	0.11–0.29		
PSO and income Q5									0.20***	0.11–0.29		
PsA and income Q1									−0.03	−0.21–0.15		
PsA and income Q2									−0.04	−0.21–0.13		
PsA and income Q4									0.13	−0.05–0.30		
PsA and income Q5									0.20**	0.02–0.38		
Born outside a Nordic country^b^	−0.03	−0.07 to 0.01	−0.04**	−0.07 to 0.01	−0.04**	−0.08 to 0.00	−0.04**	−0.08 to −0.01	−0.04**	−0.08 to 0.01	−0.05**	−0.10 to 0.01
PSO/PsA and country of birth^f^												
PSO and born outside a Nordic country											0.09*	−0.01 to 0.19
PsA and born outside a Nordic country											−0.16	−0.40 to 0.08
Observations	54,313		54,313		54,313		54,313		54,313		54,313	
R-squared	0.44		0.53		0.54		0.53		0.53		0.53	

*** *p* < 0.001, ** *p* < 0.05, * *p* < 0.1

^a^The dataset is matched for sex, age and residential area, and each matched pair is handled as a dummy variable and absorbed in the model

^b^Reference categories (Ref.) are referents, no morbidity, 10–12 years of education, income level 3 and born in a Nordic country. Ref. = 0

^c^Metabolic disease = ICD-10 group E00–E90, mental disorder = ICD-10 group F00–F99, circulatory disease = ICD-10 group I00–I99

^d^Reference category is referents with 10–12 years of education. Ref. = 0

^e^Reference category is referents with income quintile 3. Ref. = 0

^f^Reference category is referents born in a Nordic country. Ref. = 0

The income gradient worked in two directions. Both those with income below and above median were less likely to use primary care, secondary outpatient care and inpatient care, with two exceptions where income quintile 2 had a higher probability of use [non-physician professionals in secondary outpatient care (Model 1 in Table 4) and inpatient care (Model 1 in Table 5)]. Overall, these results were valid both with and without additional morbidity variables, but it is noteworthy that in Model 2, the significant association between the probability of physician use in secondary care and income in quintiles 4 and 5 disappeared (Table 4), as did the significant association between non-physician use in secondary outpatient care and income quintile 2.

Individuals born outside a Nordic country were significantly more likely to use non-physician professionals in primary care (Table 3), and the reverse was observed for inpatient care (Table 5).

We did not use the interaction models (Models 3–6) in the analysis of the probability of healthcare use. As the material was stratified in many levels and few individuals had zero healthcare use (see Supplementary Table S1), there were too few values in different data cells for analysis.

### Healthcare costs

Table 6 shows the *β*-coefficients and 95% confidence intervals for the annual mean healthcare costs across PSO/PsA, additional morbidity variables and socioeconomic/demographic variables conditional on positive healthcare use at least once during the 4-year study period (99% of the PSO/PsA patients and 96% of the referents). Model 1 shows that PSO and PsA were significantly positively associated with healthcare costs: +106% for PsA (*β* = 1.06, 95% CI 1.00–1.12) and +46% for PSO (*β* = 0.46, 95% CI 0.43–0.49) compared with the referents. From Model 2, it is seen that the presence of other morbidities was positively associated with healthcare costs. The most distinct association was noted for the presence of circulatory disease: +77% (*β* = 0.77, 95% CI 0.74–0.80).

In Model 1, low education (0–9 years) was significantly associated with higher healthcare costs than for medium education (10–12 years), while the reverse was observed for high education. The significant associations disappeared when we added morbidity variables (Model 2 in Table 6).

Overall, income showed a bell-shaped relationship to healthcare costs, with quintiles 2 and 3 having the highest mean annualized cost (Models 1–6 in Table 6). Higher income (quintiles 4 and 5) was highly associated with lower healthcare costs, as was the low income (quintile 1).

Some heterogeneity with respect to education and income was seen among patients with PSO and PsA (Models 4 and 5 in Table 6). While low education overall was associated with higher costs, the marginal effect of PSO and PsA in interaction with low education was negative (−0.12, 95% CI −0.18 to −0.05; −0.22, 95% CI −0.39 to −0.10; Model 4). The resulting total effect of PSO and PsA on the mean annualized healthcare costs showed significantly lower costs among those with low education than among those with medium education (PSO *p* = 0.04; PsA *p* = 0.01). The total effect of PSO and PsA and low education was 0.32 (95% CI 0.27–0.37) and 0.82 (0.71–0.92), respectively, which is less than the effect only of PSO and PsA in Model 2. For PsA, that is almost double the costs than for the reference category (referents with 10–12 years of education). For the total effect of education for each stratum within the PSO and PsA groups, see Table S4 in the Online Supplementary Material.

The bell-shaped pattern of income quintiles was preserved, but to a lesser extent when accounting for interactions of the presence of PSO/PsA and income (Model 5 in Table 6). The “top of the bell” was found in income quintile 2. Including the interaction terms for PSO/PsA and income increased the base effect of disease in income quintiles 4 and 5 for PSO and in income quintile 5 for PsA. However, this increase was counteracted by the negative effect found in income quintiles 4 and 5. The resulting total effect of PSO and PsA on the mean annualized healthcare costs preserved a tendency for lower healthcare costs among those with low and high incomes, particularly in the PsA group (see Table S5 in the Online Supplementary Material).

We also performed a sensitivity analysis of Model 2 in Table 6 (the results are presented in Table S3 in the Online Supplementary Material) that also included individuals with zero healthcare use (*n* = 1819). In comparison, the results of the regression, including the total study population, the coefficients for PSO/PsA and the additional morbidities, were higher than the results of Model 2 in Table 6. The direction of the coefficients for education and income showed the same pattern as in Model 2 but deviated slightly in size. Noteworthy was the significant association between being born outside a Nordic country and lower healthcare costs in the sensitivity model.

## Discussion

In this study, we explored the influence of socioeconomic and demographic factors on healthcare use and healthcare costs, controlling for need as measured by physician-confirmed presence of the chronic diseases PSO/PsA and additional morbidity. Consistent with hypotheses, the main findings were that the presence of PSO and PsA is associated with an increased probability for healthcare use across all types of healthcare service levels, and with greater healthcare costs. Furthermore, income, and to some extent education, had significant effects on the probability of healthcare use.

To analyze the robustness of education and income effects on healthcare use and costs, we also controlled for additional morbidity variables measured by the presence of metabolic, mental and circulatory diseases. When controlling for both PSO/PsA and additional morbidity variables, socioeconomic and demographic disparities in healthcare use still remained. One notable finding was that PSO and PsA patients with low education had significantly lower healthcare costs than patients with middle and high education. Furthermore, the overall effect of income was bell-shaped, i.e., those with mid-income (quintiles 2 and 3) had higher use than those with both low and high income (Model 3 in Table 6). This pattern was also found in the PSO and PsA groups (Model 5 in Table 6), but to a lesser extent. Our interpretation of this finding is that individuals suffering from a chronic disease are within the healthcare services system. In their case, decisions by a physician and other healthcare personnel govern costs, while demand-side individual socioeconomic and demographic factors may play a less important role in the healthcare use. In contrast, patient groups without a diagnosed chronic disease may not have regular healthcare contact that facilitates access.

Although the literature on the relationship between socioeconomic factors and healthcare use disparities is extensive, few of the studies are recent. As both treatment patterns and healthcare system designs change over time, there are potential new conditions for policy-related research, which should encourage more updated studies. However, existing studies do indicate some evidence that socioeconomic factors influence healthcare use in various ways depending on the healthcare service level. While the patient has the initiative when consulting primary care, the primary care physician plays a role as gatekeeper for the subsequent referrals and visits to secondary outpatient care, and the patient’s role in the decision about inpatient care is likely to be limited. A number of studies with slightly different designs and also different definitions of need from the ones in our study have consistently shown higher use of primary care services among lower income groups and higher use of specialist services among higher income groups in a number of countries in Europe and North America [1, 10, 14, 21, 42]. A Norwegian study supported the finding of higher use of secondary outpatient care among higher income individuals, but did not find any income-related influence on primary care use [39].

In contrast to these studies, our results showed that, for both PSO and PsA groups and across all healthcare service types, individuals in income quintiles 2 and 3 were more likely to use healthcare and they had higher costs than those with lower or higher income had. To what extent the results are linked to financial barriers in quintile 1 or other causes is beyond the scope of this study, but such barriers do not seem to impact on observed use or costs in quintiles 2 and 3. A priori, we assumed low financial barriers, since the co-payment paid by the patient in Sweden is low and is subject to a high cost ceiling. However, a recent Swedish study found some evidence for self-reported refraining from healthcare for financial reasons among more vulnerable socioeconomic groups [43]. It is worth noting that, in our study, a number of those in income quintile 1 were registered with very low income.

Other explanations for our findings may be that those with lower and higher income perceive their health status to be better than those with median income do, or that their preferences for healthcare are different. Socioeconomic and demographic variables could be correlated with differences in the individuals’ preferences for healthcare use, but other data would be needed to address such underlying factors.

Any use, as opposed to volume of use, was chosen as the unit of analysis because there is reason to believe that the characteristics of the individual are of primary importance for the initial healthcare consultation, whereas the physician determines the volume of use [9, 10, 13]. In an older Swedish study on self-reported information on physician consultations, it was concluded that low income was associated with a lower probability of any use of physician care, but not with the frequency of consultation. However, the drawback in this study was the non-differentiation between consultations in primary care versus secondary outpatient care [13].

In our study, education seemed to be of significant importance for healthcare use, while it had less influence on healthcare costs among those with healthcare use. Furthermore, low income was associated with a lower probability of healthcare use than for those with median income, but low income (quintile 1) patients had lower costs than referents with median income had.

An interpretation of our results from an equity perspective would be that the principle of horizontal equity in healthcare use is violated in our study population. After controlling for the presence of PSO/PsA and additional morbidity, there was still some effect of socioeconomic variables on both the probability of healthcare use and healthcare costs. There were also some differences related to the type of healthcare service level and the type of healthcare provider. These differences imply that simple categorical conclusions regarding the presence of horizontal inequity in healthcare use open up further questions, such as how to aggregate inequity observed at different healthcare service levels and how different components of inequity should be ranked.

A major strength of this study was the use of individual-level data from a large population over 4 years, capturing even rare consumers of healthcare [44] and facilitating robust cost estimates. A previous validation study from our group has shown limited misclassification of patients with PSO and PsA in the SHR (i.e., high positive predicted value for the diagnostic codes) [23], which suggests that problems associated with misclassification are small in the present study.

This study has some limitations that should be addressed. We included only individuals consulting a healthcare provider for their PSO or PsA problems during the 10-year inclusion period. However, the long inclusion period reduces the concern about missing infrequent healthcare users, such as individuals with mild symptoms. Furthermore, there may be omitted-variables bias. Not including total morbidity or self-reported perceived health, the models may over- or underestimate the effect of socioeconomic factors on healthcare use and cost because of differences in health across different socioeconomic gradients. The previous literature has pointed to the difficulties associated with defining and measuring need in studies analyzing the impact of socioeconomic variables on healthcare use while controlling for need [45]. However, one consistent finding seems to be that the more extensive the specification used to define need is, the less likely it is to find evidence of inequity across socioeconomic variables [14, 46, 47]. Our results support this finding, with part of the PSO/PsA effect appearing to be due to the additional morbidities that are common in the general population and which are also known comorbidities of PSO and PsA.

The case-control design of the study with three referents for each case implies that the study population’s characteristics were not representative of the characteristics of the general population. Our referent cohort included people of all ages above 19 years, but the emphasis was on people of higher ages. Nevertheless, our main interest lay in understanding whether differences exist between people with PSO/PsA and a similar population without those diseases.

This research can be seen as one contribution to the overall understanding of how to improve health and healthcare services for patients with PSO and PsA carried out by different stakeholders. In 2014, PSO was included in the WHO’s strategy work on non-communicable chronic diseases [48], and in Sweden, the National Board of Health and Welfare was recently assigned by the government to study psoriasis and examine what the needs are and what efforts could improve the care for these patients [49]. One of the reasons for these newly initiated efforts in the field of PSO and PsA is the indication of unmet needs related to access to care and coordination between different specialists [48, 50, 51].

This study adds to the knowledge of what the disparities in healthcare use are and in what way they are systematically related to socioeconomic and demographic variables. This knowledge can assist in designing adequate policies that improve health for people with chronic disease.

In conclusion, our results indicate that socioeconomic disparities remain, especially related to income, in the pattern of healthcare use among a cohort of patients and referents after controlling for the presence of PSO and PsA and other common morbidities. Having PSO or PsA did not seem to entail any additional negative impact from education or income. Instead, having a chronic disease such as PSO or PsA appeared to smooth out the bell-shaped effect of income.

## Electronic supplementary material

Below is the link to the electronic supplementary material.

Supplementary material 1 (DOCX 32 kb)

## References

[1] Devaux M (2015). Income-related inequalities and inequities in health care services utilisation in 18 selected OECD countries. Eur. J. Health Econ..

[2] Glied S, Smith PC (2011). The Oxford handbook of health economics.

[3] LeGrand J (1991). Equity and choice. An essay in economics and applied philosophy.

[4] Asadi-Lari M, Packham C, Gray D (2003). Need for redefining needs. Health and quality of life outcomes.

[5] Culyer AJ, Wagstaff A (1993). Equity and equality in health and health care. J. Health Econ..

[6] Tsuchiya A, Dolan P (2009). Equality of what in health? Distinguishing between outcome egalitarianism and gain egalitarianism. Health Econ..

[7] Shadmi E, Balicer RD, Kinder K, Abrams C, Weiner JP (2011). Assessing socioeconomic health care utilization inequity in Israel: impact of alternative approaches to morbidity adjustment. BMC public health.

[8] Agerholm J, Bruce D, Ponce de Leon A, Burstrom B (2013). Socioeconomic differences in healthcare utilization, with and without adjustment for need: an example from Stockholm. Sweden. Scand J Public Health.

[9] Alberts JF, Sanderman R, Eimers JM, van den Heuvel WJ (1997). Socioeconomic inequity in health care: a study of services utilization in Curacao. Soc. Sci. Med. (1982).

[10] Asada Y, Kephart G (2007). Equity in health services use and intensity of use in Canada. BMC Health Serv. Res..

[11] Nordin M, Dackehag M, Gerdtham UG (2013). Socioeconomic inequalities in drug utilization for Sweden: evidence from linked survey and register data. Soc. Sci. Med..

[12] Fleurbaey M, Schokkaert E, Pauly M, McGuire T, Barros P (2011). Equity in health and health care. Handbook of health economics.

[13] Gerdtham UG (1997). Equity in health care utilization: further tests based on hurdle models and Swedish micro data. Health Econ..

[14] Morris S, Sutton M, Gravelle H (2005). Inequity and inequality in the use of health care in England: an empirical investigation. Soc. Sci. Med. (1982).

[15] Hjern A, Haglund B, Persson G, Rosen M (2001). Is there equity in access to health services for ethnic minorities in Sweden?. Eur. J. Pub. Health.

[16] Layte R, Nolan A (2015). Income-related inequity in the use of GP services by children: a comparison of Ireland and Scotland. Eur. J. Health Econ..

[17] Masseria C, Giannoni M (2010). Equity in access to health care in Italy: a disease-based approach. Eur. J. Pub. Health.

[18] Vallejo-Torres L, Morris S (2013). Income-related inequity in healthcare utilisation among individuals with cardiovascular disease in England—accounting for vertical inequity. Health Econ..

[19] Geale K, Henriksson M, Schmitt-Egenolf M (2015). Evaluating equity in psoriasis healthcare: a cohort study of the impact of age on prescription biologics. Br. J. Dermatol..

[20] Orueta JF, Garcia-Alvarez A, Alonso-Moran E, Vallejo-Torres L, Nuno-Solinis R (2013). Socioeconomic variation in the burden of chronic conditions and health care provision—analyzing administrative individual level data from the Basque Country, Spain. BMC Public Health.

[21] van Doorslaer, E., Masseria, C., Koolman, X.: Inequalities in access to medical care by income in developed countries. CMAJ: Canadian Medical Association journal = journal de l’Association medicale canadienne **174**(2), 177–183 (2006). doi:10.1503/cmaj.050584 10.1503/cmaj.050584 PMC1329455 16415462

[22] Helliwell PS, Ruderman EM (2015). Natural history, prognosis, and socioeconomic aspects of psoriatic arthritis. Rheum. Dis. Clin. North Am..

[23] Lofvendahl S, Theander E, Svensson A, Carlsson KS, Englund M, Petersson IF (2014). Validity of diagnostic codes and prevalence of physician-diagnosed psoriasis and psoriatic arthritis in southern Sweden—a population-based register study. PLoS One.

[24] Wallenius M, Skomsvoll JF, Koldingsnes W, Rodevand E, Mikkelsen K, Kaufmann C, Kvien TK (2009). Work disability and health-related quality of life in males and females with psoriatic arthritis. Ann. Rheum. Dis..

[25] Sweden, P.H.A.o.: Folkhälsa i Sverige. Årsrapport 2014 [Public Health in Sweden. Annual report 2014]. Public Health Agency of Sweden. http://www.folkhalsomyndigheten.se/publicerat-material/publikationer/Folkhalsan-i-Sverige-arsrapport-2014 (2014) 20160712. Accessed 12 July 2016

[26] Husni ME (2015). Comorbidities in psoriatic arthritis. Rheum. Dis. Clin. North Am..

[27] Kimball AB, Guerin A, Tsaneva M, Yu AP, Wu EQ, Gupta SR, Bao Y, Mulani PM (2011). Economic burden of comorbidities in patients with psoriasis is substantial. J. Eur. Acad Dermatol. Venereol..

[28] Bhole VM, Choi HK, Burns LC, Vera Kellet C, Lacaille DV, Gladman DD, Dutz JP (2012). Differences in body mass index among individuals with PsA, psoriasis, RA and the general population. Rheumatology (Oxford England).

[29] Phan C, Sigal ML, Lhafa M, Barthelemy H, Maccari F, Esteve E, Reguiai Z, Perrot JL, Chaby G, Maillard H, Begon E, Alexandre M, Toussaint P, Bastien-Jacquin M, Bravard P, Sauque E, de Quatrebarbes J, Pfister P, Beauchet A, Mahe E (2016). Metabolic comorbidities and hypertension in psoriasis patients in France. Comparisons with French national databases. Ann. Dermatol. Venereol..

[30] Gulati AM, Semb AG, Rollefstad S, Romundstad PR, Kavanaugh A, Gulati S, Haugeberg G, Hoff M (2016). On the HUNT for cardiovascular risk factors and disease in patients with psoriatic arthritis: population-based data from the Nord-Trondelag Health Study. Ann. Rheum. Dis..

[31] Dubreuil M, Rho YH, Man A, Zhu Y, Zhang Y, Love TJ, Ogdie A, Gelfand JM, Choi HK (2014). Diabetes incidence in psoriatic arthritis, psoriasis and rheumatoid arthritis: a UK population-based cohort study. Rheumatology (Oxford, England).

[32] Boehncke WH, Kirby B, Fitzgerald O, van de Kerkhof PC (2013). New developments in our understanding of psoriatic arthritis and their impact on the diagnosis and clinical management of the disease. J. Eur. Acad. Dermatol. Venereol..

[33] Lofvendahl S, Petersson IF, Theander E, Svensson A, Zhou C, Steen Carlsson K (2016). Incremental costs for psoriasis and psoriatic arthritis in a population-based cohort in Southern Sweden: is it all psoriasis-attributable morbidity?. J. Rheumatol..

[34] Kjellsson G, Clarke P, Gerdtham UG (2014). Forgetting to remember or remembering to forget: a study of the recall period length in health care survey questions. J. Health Econ..

[35] Swedish Health Account. http://www.scb.se/en_/Finding-statistics/Statistics-by-subject-area/National-Accounts/National-Accounts/System-of-Health-Accounts-SHA/ (2010). Accessed 25 November 2013

[36] Wettermark B, Hammar N, Fored CM, Leimanis A, Otterblad Olausson P, Bergman U, Persson I, Sundstrom A, Westerholm B, Rosen M (2007). The new Swedish Prescribed Drug Register—opportunities for pharmacoepidemiological research and experience from the first six months. Pharmacoepidemiol. Drug Saf..

[37] Mihaylova B, Briggs A, O’Hagan A, Thompson SG (2011). Review of statistical methods for analysing healthcare resources and costs. Health Econ..

[38] van Doorslaer E, Wagstaff A, van der Burg H, Christiansen T, De Graeve D, Duchesne I, Gerdtham UG, Gerfin M, Geurts J, Gross L, Hakkinen U, John J, Klavus J, Leu RE, Nolan B, O’Donnell O, Propper C, Puffer F, Schellhorn M, Sundberg G, Winkelhake O (2000). Equity in the delivery of health care in Europe and the US. J. Health Econ..

[39] Vikum E, Krokstad S, Westin S (2012). Socioeconomic inequalities in health care utilisation in Norway: the population-based HUNT3 survey. Int. J. Equity Health.

[40] stcox—Cox proportional hazards model. http://www.stata.com/manuals13/ststcox.pdf (2014). Accessed 24 March 2016

[41] areg—Linear regression with a large dummy-variable set. http://www.stata.com/manuals13/rareg.pdf (2014). Accessed 24 March 2016

[42] Dunlop S, Coyte PC, McIsaac W (2000). Socio-economic status and the utilisation of physicians’ services: results from the Canadian National Population Health Survey. Soc. Sci. Med. (1982).

[43] Molarius A, Simonsson B, Linden-Bostrom M, Kalander-Blomqvist M, Feldman I, Eriksson HG (2014). Social inequalities in self-reported refraining from health care due to financial reasons in Sweden: health care on equal terms?. BMC Health Ser. Res..

[44] Icen M, Crowson CS, McEvoy MT, Gabriel SE, Maradit Kremers H (2008). Potential misclassification of patients with psoriasis in electronic databases. J. Am. Acad. Dermatol..

[45] Gravelle H, Morris S, Sutton M, Jones AM (2006). Economic studies of equity in the consumption of health care. The Elgar companion to health economics.

[46] Wagstaff, A., Doorslaer, E.: Equity in health care finance and delivery. In: Culyer, A.J., Newhouse, J.P. (eds.) Handbook of health economics, vol. 1. pp. 1803–1862. Elsevier (2000)

[47] Goddard M, Smith P (2001). Equity of access to health care services: theory and evidence from the UK. Soc. Sci. Med. (1982).

[48] World Health Organization: WHO resolution on psoriasis. http://apps.who.int/gb/ebwha/pdf_files/WHA67/A67_R9-en.pdf (2014). Accessed 1 June 2016

[49] Förstudie om psoriasis [Pilot study on psoriasis]. http://www.socialstyrelsen.se/nyheter/2015maj/socialstyrelsengorforstudieomendometrios (2015). Accessed 1 June 2016

[50] Bhutani T, Wong JW, Bebo BF, Armstrong AW (2013). Access to health care in patients with psoriasis and psoriatic arthritis: data from National Psoriasis Foundation survey panels. JAMA Dermatol..

[51] Uttjek M, Dufaker M, Nygren L, Stenberg B (2005). Psoriasis care consumption and expectations from a gender perspective in a psoriasis population in northern Sweden. Acta dermato-Venereologica.

