# Supplementary material for: Income disparities in healthcare use remain after controlling for healthcare need: evidence from Swedish register data on psoriasis and psoriatic arthritis
Source: Eur J Health Econ. 2017 May 19;19(3):447–62. doi: 10.1007/s10198-017-0895-5 (PMC5978916; doi:10.1007/s10198-017-0895-5)
Supplement: Supplementary file 1 — Supplementary material 1 (DOCX 32 kb) [file 10198_2017_895_MOESM1_ESM.docx]

**Online Supplementary Material**

**Authors**

Sofia Löfvendahl, MSc^1,2^ Anna Jöud, PhD, MPH, ^2,7^ Ingemar F. Petersson, PhD, MD^1,2^ Elke Theander, PhD, MD^3^ Åke Svensson, PhD, MD^4^ Katarina Steen Carlsson, PhD^1,5^

**Affiliations**

**Affiliations**

^1^ Lund University, Faculty of Medicine, Department of Clinical Sciences Lund, Orthopedics, Lund, Sweden

^2^ ERC Syd Region Skåne, Skåne University Hospital, Lund, Sweden

^3^ Department of Rheumatology, Skåne University Hospital, Malmö, Sweden

^4^ Department of Dermatology, Skåne University Hospital, Malmö, Sweden

^5^ Department of Clinical Sciences, Malmö, Lund University, Skåne University Hospital, Malmö, Sweden

^7^ Division of occupational and environmental medicine, Lund University, Lund, Sweden

**In the supplementary descriptive material PSO patients and PsA patients are presented together.**

| **Table of contents** | |
| --- | --- |
| **Table S1**. | Percentage (%) of PSO/PsA patients and referents with at least one visit/inpatient day during follow-up 2008-2011 across education, income, country of birth and residential area. |
| **Table S2**. | Mean annual healthcare costs during follow-up 2008-2011 across education, income, country of birth and residential area. |
| **Table S3**. | Sensitivity analysis of regression Model 2 in Table 4 in the main manuscript. |
| **Table S4** | Total effect of PSO/PsA, education and interaction of PSO/PsA and education on mean annualized healthcare costs |
| **Table S5** | Total effect of PSO/PsA, income and interaction of PSO/PsA and income on mean annualized healthcare costs |

| **Table S1**. Percentage (%) of PSO/PsA patients and referents with at least one visit/inpatient day during follow-up 2008-2011 across morbidity, education, income, country of birth and residential area. | | | | | | | | | | | | | | | |
| --- | --- | --- | --- | --- | --- | --- | --- | --- | --- | --- | --- | --- | --- | --- | --- |
|  | **Primary care** | | | | |  | **Secondary care** | | | | |  | | **Inpatient care** | |
|  | Physician | |  | Other personnel | |  | Physician | |  | Other personnel | |  | | Physician | |
| **Variable** | PSO/PsA patients | Referents |  | PSO/PsA patients | Referents |  | PSO/PsA patients | Referents |  | PSO/PsA patients | Referents |  | PSO/PsA patients | | Referents |
|  |  |  |  |  |  |  |  |  |  |  |  |  |  | |  |
| **Morbidity** |  |  |  |  |  |  |  |  |  |  |  |  |  | |  |
| Metabolic disease | 98 | 98 |  | 92 | 92 |  | 97 | 95 |  | 82 | 75 |  | 58 | | 56 |
| Mental disorders | 98 | 97 |  | 90 | 87 |  | 97 | 94 |  | 77 | 68 |  | 53 | | 51 |
| Circulatory disease | 98 | 98 |  | 92 | 90 |  | 97 | 95 |  | 77 | 69 |  | 59 | | 57 |
| **Education** |  |  |  |  |  |  |  |  |  |  |  |  |  | |  |
| Low (≤9 years) | 95 | 91 |  | 87 | 82 |  | 93 | 86 |  | 69 | 57 |  | 47 | | 41 |
| Moderate (10-12 years) | 93 | 88 |  | 84 | 79 |  | 91 | 82 |  | 64 | 50 |  | 35 | | 28 |
| High (≥12 years) | 89 | 82 |  | 81 | 77 |  | 90 | 81 |  | 63 | 48 |  | 30 | | 25 |
| **Income** |  |  |  |  |  |  |  |  |  |  |  |  |  | |  |
| Quintile 1 (Low) | 91 | 82 |  | 85 | 75 |  | 90 | 78 |  | 68 | 52 |  | 43 | | 34 |
| Quintile 2 | 95 | 92 |  | 88 | 84 |  | 94 | 89 |  | 73 | 62 |  | 51 | | 44 |
| Quintile 3 | 95 | 90 |  | 88 | 85 |  | 93 | 87 |  | 68 | 55 |  | 38 | | 34 |
| Quintile 4 | 92 | 88 |  | 83 | 79 |  | 90 | 82 |  | 60 | 46 |  | 28 | | 23 |
| Quintile 5 (High) | 90 | 83 |  | 78 | 74 |  | 89 | 78 |  | 57 | 42 |  | 25 | | 22 |
| **Country of birth** |  |  |  |  |  |  |  |  |  |  |  |  |  | |  |
| Nordic | 93 | 87 |  | 84 | 79 |  | 91 | 83 |  | 65 | 51 |  | 39 | | 32 |
| Non-Nordic | 90 | 81 |  | 81 | 74 |  | 88 | 77 |  | 65 | 49 |  | 33 | | 28 |
|  | | | | | | | | | | | | | | | |

| **Table S2**. Mean annual healthcare costs during follow-up 2008-2011 across morbidity, education, income, country of birth and residential area. | | |
| --- | --- | --- |
|  | **Total healthcare costs, mean (sd)^1^** | |
|  | PSO/PsA patients ^¤^ | Referents ^¤¤^ |
| **Morbidity** |  |  |
| Metabolic disease | 7949 (11395) | 6166 (9919) |
| Mental disorders | 6743 (10285) | 5399 (8999) |
| Circulatory disease | 7621 (11168) | 5774 (9171) |
| **Education** |  |  |
| Low (≤9 years) | 5792 (9378) | 4204 (7840) |
| Moderate (10-12 years) | 4571 (8631) | 2793 (6115) |
| High (≥12 years) | 3774 (6972) | 2316 (4976) |
| **Income** |  |  |
| Quintile 1 (Low) | 5359 (9819) | 3528 (7452) |
| Quintile 2 | 6725 (10246) | 4660 (8536) |
| Quintile 3 | 4937 (8988) | 3230 (6373) |
| Quintile 4 | 3512 (6457) | 2161 (4559) |
| Quintile 5 (High) | 3306 (6400) | 1938 (4225) |
| **Country of birth** |  |  |
| Nordic | 5022 (8856) | 3315 (7017) |
| Non-Nordic | 4481 (10076) | 2906 (7491) |
| ^1^ Total healthcare costs include costs due to drug use in addition to cost due to visits within primary care, secondary outpatient care and inpatient care.  All costs are expressed in EUR in prices of 2011.  ^¤^Number of PSO/PsA individuals with any healthcare costs during 2008-2011=14,318  ^¤¤^Number of referent individuals with any healthcare costs during 2008-2011=41,663 | | |

Sensitivity analysis of regression Model 2 in Table 4 in the main manuscript.

| **Table S3**. Linear regression of factors influencing mean annual healthcare costs during the period 2008-2011 (costs are in logarithm form). In regression. In this regression we have included also the individuals with zero healthcare contacts during the study period. | | |
| --- | --- | --- |
| **Variabels^#^** | **Model 2** | |
|  | β | 95% CI |
|  |  |  |
| Presence of PSO/PsA |  |  |
| No presence (Ref) |  |  |
| PSO ^¤^ | 0.47*** | 0.43-0.50 |
| PsA | 1.07*** | 1.00-1.13 |
|  |  |  |
| Metabolic disease^¤, £^ | 0.61*** | 0.57-0.65 |
| Mental disorders^¤, £^ | 0.85*** | 0.81-0.89 |
| Circulatory disease^¤, £^ | 0.95*** | 0.91-0.99 |
|  |  |  |
|  |  |  |
| Education^¤^ |  |  |
| 0-9 years | 0.01 | -0.03 - 0.05 |
| 10-12 years (Ref) |  |  |
| >12 years | -0.07*** | -0.11 - -0.03 |
|  |  |  |
| Income ^¤^ |  |  |
| Quintile 1 (Low) | -0.28*** | -0.33 - -0.23 |
| Quintile 2 | 0.01 | 0.04-0.05 |
| Quintile 3 (Ref) |  |  |
| Quintile 4 | -0.04* | -0.09 - 0.00 |
| Quintile 5 (High) | -0.11*** | -0.16 - -0.06 |
|  |  |  |
| Born outside a Nordic country^¤^ | -0.13*** | -0.17 - 0.08 |
|  |  |  |
| Observations | 55,791 | |
| R-squared | 0.34 | |
| ^#^The dataset is matched for sex, age and residential area. Regressions controlled for matched pair fixed effects using Stata command areg.  ^¤^Reference categories are referents, no morbidity education 10-12 years, income level 3 and born in a Nordic country. Ref=0  ^£^Metabolic disease =ICD10 group E00-E90. Mental disorders=ICD-10 group F00-F99. Circulatory disease=ICD-10 group I00-I99  ^¤¤¤^Reference category is referents with 10-12 years of education. Ref=0  ^¤¤¤¤^Reference category is referents with income quintile (Q3). Ref=0  ^¤¤¤¤¤^Reference category is referents born in a Nordic country. Ref=0  ***p<0.001, **p<0.05, *p<0.1  This regression analysis includes 20 more individuals compared to the corresponding Cox-regression analysis in the main manuscript. In the Cox regression individuals entering and exiting the study on the same day are excluded (n=20) | | |

| **Table S4.** Total effect of PSOand PsA by level of education on mean annualized healthcare costs calculated from base and interaction effects according to Model 4 in Table 4 in main text. | | |
| --- | --- | --- |
| **PSO** | β | 95% CI |
| PSO and 0-9 years | 0.32*** | 0.27-0.38 |
| PSO and 10-12 years (Ref) | 0.39*** | 0.35-0.43 |
| PSO and >12 years | 0.38*** | 0.30-0.44 |
| **PsA** |  |  |
| PsA and 0-9 years | 0.82*** | 0.71-0.92 |
| PsA and 10-12 years (Ref) | 0.98*** | 0.91-1.01 |
| PsA and >12 years | 0.95*** | 0.80-1.10 |

Ref=Reference category

***p<0.001, **p<0.05, *p<0.1

| **Table S5.** Total effect of PSO and PsA by income quintile on mean annualized healthcare costs calculated from base and interaction effects according to Model 5 in Table 4 in main text. | | |
| --- | --- | --- |
| **PSO** | β | 95% CI |
| PSO and income Q1 | 0.28*** | 0.21-0.35 |
| PSO and income Q2 | 0.31*** | 0.25-0.38 |
| PSO and income Q3 (Ref) | 0.26*** | 0.20-0.32 |
| PSO and income Q4 | 0.31*** | 0.25-0.35 |
| PSO and income Q5 | 0.22*** | 0.15-0.28 |
| **PsA** |  |  |
| PsA and income Q1 | 0.74*** | 0.61-0.88 |
| PsA and income Q2 | 0.89*** | 0.77-1.00 |
| PsA and income Q3 (Ref) | 0.87*** | 0.75-0.99 |
| PsA and income Q4 | 0.86*** | 0.73-0.98 |
| PsA and income Q5 | 0.83*** | 0.70-0.97 |

Ref=Reference category

***p<0.001, **p<0.05, *p<0.1
